# Labor market costs for long-term family caregivers: the situation of caregivers of persons with spinal cord injury in Switzerland

**DOI:** 10.1186/s12913-023-09565-7

**Published:** 2023-06-22

**Authors:** Monica Ruoss, Mirjam Brach, Diana Pacheco Barzallo

**Affiliations:** 1 Faculty of Health Sciences and Medicine, University of Lucerne, Frohburgstrasse 3, 6002 Lucerne, Switzerland; 2 Swiss Paraplegic Research, Guido A. Zäch Str. 4, 6207 Nottwil, Switzerland; 3 Center for Rehabilitation in Global Health Systems, WHO Collaborating Center, Lucerne, Switzerland

**Keywords:** Family caregivers, Disability, Care needs, Labor market costs

## Abstract

**Background:**

Family members are key in the provision of care to persons facing disability. To undertake the role as caregivers, they face many costs, being the setback in the labor market one of the most relevant.

**Methods:**

We analyze comprehensive data from long-term family caregivers of persons with spinal cord injury (SCI) in Switzerland. Using information about their working situation before and after becoming caregivers, we estimated the reduction in working hours and the associated income loss.

**Results:**

On average, family caregivers reduced their working hours by about 23% (8.4 h per week), which has a monetary value of CHF 970 per month (EUR 845). Women, older caregivers, and less educated caregivers have a much higher opportunity cost in the labor market: CHF 995 (EUR 867), CHF 1,070 (EUR 932), and CHF 1,137 (EUR 990) respectively. In contrast, family members who care for a person that works have a much lower impact on their working status, CHF 651 (EUR 567). Interestingly, the reduction in their working time is only a third of the extra work they face as caregivers.

**Conclusion:**

Health and social systems rely on the unpaid work of family caregivers. To guarantee their long-term involvement, family caregivers need to be recognized for their work and potentially compensated. Without family caregivers, it is very unlikely societies can cope with the increasing need for care, as professional services are limited and expensive.

## Introduction

Health systems are heavily reliant on the work undertaken by family caregivers, work that is generally unpaid [1]. Nevertheless, considering their work as a “free resource” is inadequate, as it entails many costs, being the setback in the labor market one of the most important and difficult to compensate for [2, 3, 4]. In general, family members reduce their working hours or stop working altogether to cope with the needs of care in the family [5]. And when households cannot afford the reduction in income, family members add caring responsibilities to their working schedule, which is not always the most desirable arrangement neither for the caregiver nor for the cared-for person [6, 7].

The objective of this paper is to estimate the opportunity cost in the labor market for long-term family caregivers in Switzerland. We focus on caregivers of persons with spinal cord injury (SCI), a group that reports one of the highest burdens compared to other caregivers’ groups as they spend numerous hours per day, for years, caring for a relative [8]. SCI is a complex condition that combines a high level of physical impairment (paraplegia/tetraplegia) with a series of comorbidities. Family caregivers of this group undertake a series of tasks that go from eating and drinking to transportation, to support with bowel and bladder management [8, 9]. In fact, SCI is a high-needs and high-cost health condition that is generally the result of an accident [10–12]. This implies that family members become caregivers with any anticipation and possibility of adjustment. 

In Switzerland, long-term care does not directly support family caregivers, but care and support are provided based on the needs of a person. Professional home care is reimbursed as long as a doctor prescribes the need for the service and it is undertaken by a professional caregiver. Non-medical tasks, such as housekeeping or support with paperwork are never reimbursed [13, 14]. Thus, family members cannot receive any kind of compensation, even when their role is deemed essential for the provision of professional home care, i.e. family caregivers play a key role in the interaction between professional care services and the cared-for person [13]. Nevertheless, in some cases, family caregivers can go into specific training to become professional caregivers and be able to charge for their services.

The role of family caregivers is becoming more relevant as demographic dynamics, with the rise in non-communicable diseases, forecast a sharp increase in the number of people requiring care [15, 16]. Policy makers need to find ways to support family caregivers to guarantee their involvement in the caregiving process. Otherwise, health and social systems will not be able to cope with the population's needs as the provision of professional home care is expensive and requires an important number of trained workers [17–19].

To support family caregivers, health systems can either compensate them for their caregiving time, or support with their caring responsibilities to make their work less cumbersome. Nevertheless, in either case, it is essential to first understand what involves caregiving, as well as the trade-offs family members face. To date, there is an increasing interest to include economic evaluations to account for the work performed by family caregivers as fiscal sustainability of the current long-term care provision is raising concerns [4, 20, 21]. Unfortunately, comprehensive data is still scarce, and existing information reduces caregiving to a number of hours of care and disregards the complexity of care. Also, family caregiving falls under the family privacy, which makes it less visible and difficult to inquire. For family members, it is difficult to disentangle the work they do as caregivers on top of their regular tasks. Additionally, there exist methodological issues because caregiving is heterogeneous and depends on the needs and characteristics of the cared-for person [22, 23]. For some health conditions, family caregivers undertake a series of tasks, some of which require some level of expertise.

Related studies estimated the economic value of family caregivers by looking at caregivers of elderly population [24], or caregivers of people with specific health conditions [25]. In general, the estimated economic value has high variations and is highly dependent on the characteristics of the cared-for person. In the case of Switzerland, there are two studies closer to ours that monetise the work undertaken by long-term family caregivers. The first study looked at caregivers of persons with Alzheimer and estimated their work in about CHF 4,608 per month (CHF 55,300 per year) [26]. A second study looked at caregivers of persons with SCI and estimated their work in about CHF 5,227 per month (62,732 per year) [27]. In the absence of family caregivers, and following the current long-term care law in Switzerland, these values should be covered mostly by the health insurances, social insurances, and with out-of-pocket payments, with important implications for the Swiss health and social systems but also for the financial situation of the households [27].

In most of the existing studies, the economic value is estimated by computing the costs of replacing the work of family caregivers with a market substitute, method known as proxy-good method. Nevertheless, defining what is the closer substitute is not simple and depends on how long-term care is organized in each county. There are few studies that estimate the economic value of family caregivers by the opportunity cost in the labor market [28, 29]. This happens because gathering data on labor market outcomes is more challenging as it requires information before and after a family member became caregiver. Existing studies overcome these challenges by comparing the work status of family caregivers to their counterparts in the general population, and monetize their work using wage rates from skills and unskilled labor [28, 30, 31]. In spite of all different methodologies, all related studies concluded that the work undertaken by family caregivers is worth many times more than what is currently spent on long-term care. The contribution of our study is that we have comprehensive information about the working situation of family members before and after they became caregivers. Also, as SCI is mostly the results of an accident, the needs for care constrained the working decisions of family members, and not the other way around. This characteristics allowed us to identify the adjustments family members undertook in their working situation to become caregivers without additional assumptions. 

## Methods

### Data

This study analysed cross-sectional data from a questionnaire launched in 2016 and closed in 2017. The questionnaire was directed to persons with SCI, or cared-for person, registered in the Swiss Spinal Cord Injury Cohort Study (SwiSCI) [32, 33]. SwiSCI is a registry of persons with SCI from the four SCI specialised clinics in Switzerland, patients associations, and wheelchair clubs. To contact the participants, the SwiSCI Study Center sent an invitation-letter to persons with SCI who were not institutionalised. The letter asked the person with SCI to forward the questionnaire to their primary family caregivers (*N*=4502). If the person with SCI did not have a family caregiver, they were asked to reply to the letter informing about it (*N*=1259). Participants had the option of completing the questionnaire of paper, online, by a phone call, or in person during an interview with a member of the research team. The inclusion criteria for participants were family caregivers, older than 18 years old, living in Switzerland, who could answer the questionnaire in one of the three official languages: German, French, or Italian [8]. From the total, 864 were identified as not eligible, and 717 participants completed and returned the questionnaire, which is a 35% response rate [8]. For the analysis, we limited the sample to all those family caregivers in working age. We excluded participants that were retired due to age, or due to a health condition, and homemakers.

The questionnaire was developed by a team of nursing experts, clinical SCI specialists, social counsellors, representatives from home care and patient organisations, health scientists, and persons with SCI. The questionnaire was developed in German, and later translated to French and Italian. The questionnaire included 138 items about the family caregiver that covered the socio-economic situation and demographics, living situation and quality of life, working and financial situation, information needs, health services utilization, personal relationships, leisure and social activities, and caregiving tasks. An additional ten questions were included about the socio-demographic characteristics as well as some information about the health conditions of the cared-for person. The items in the questionnaire were adapted from existing instruments such as the Swiss Household Panel (SHP), the Swiss Health Survey, Carers of Older People in Europe (COPE), EUROFAMCARE, Health Information National Trends Survey, and WHO-QoL [8].

In the case of the labor market situation, family caregivers were asked about their current work situation, whether they were in gainful employment, the number of working hours, type of activity, and income. Caregivers were also inquired about how caregiving changed their working situation, in terms of working hours, and type of activity before they became caregivers. 

### Data analysis

To estimate the economic value of informal caregivers, we implemented the opportunity cost method, which computes the forgone income in an alternative activity [22, 33]. To do so, we first identified those family caregivers who reported that to become family caregivers they had to adjust their working situation. More specifically, in the questionnaire, people were asked:

- *Has anything changed in relation to your gainful activity since you began to support the person with spinal cord injury?*

Answers:

1. *Nothing has changed*;
2. *Yes, I continue working with a change of employment*;
3. *Yes, I have stopped being gainfully employed*.

The opportunity cost is calculated as the differences between the working situation before minus the working situation after becoming a caregiver. Thus, when a family caregiver reported that becoming a caregiver did not change their working situation, the opportunity cost was set to zero (0). When a family caregiver reported the second or third option, the opportunity cost was either negative (-) or positive ( +), depending on how the working status has changed. A negative opportunity cost imply that becoming a caregiver translated into a reduction of the working hours/salary. In contrast, a positive opportunity cost imply that becoming a caregivers increased the working hours/salary. In fact, while most family caregivers reduced their workload outside home, others continued working or even increased their working hours.

As we only had information about whether a person worked before becoming a caregiver, and the type of activity,  we had to estimate their previous income. To do so, we used the information provided about their working conditions before they became caregivers and matched it to the General Classification of Economic Activities (NOGA). The NOGA is the Swiss version of the European Classification of Activities that sorts occupations by their main economic activity, and is commonly used for statistical purposes by insurance companies, recruitment offices, and pension funds to set premiums. Once the economic activity of the family caregiver was identified, and using their background characteristics, such as age, sex, nationality, and education it was possible to identify the median monthly salary for that activity with the tool SALARIUM.

SALARIUM is a statistical tool developed by the Federal Offices of Statistics (FSO), which compiles information about the salary distribution by economic activity. SALARIUM has data from the Swiss structural income survey (LSE), and has more than 900,000 wage statements from private sector employees [33]. The salaries are estimated using a model that considers 14 characteristics, including industry and company size as well as individual characteristics of the employees and information of the workplace [33]. For caregivers with complete information, we retrieved the median income in the market for their professional activities, and for their persons characteristics. In cases when the working characteristics of the caregiver were missing, or incomplete, we retrieved, as a reference, the lower bound salary estimate to keep a conservative approach and avoid overestimating the salaries.

Finally, as an accuracy check, we did the same process for those caregivers that had complete information, and compared the reported salaries with those estimated by SALARIUM. As the income was reported in ranges, we checked if the estimated income of SALARIUM fell within the reported range. On average, the values were very similar. In cases when the estimated salary was outside the reported range, we chose the closest value.

### Estimated forgone income

The opportunity cost in the labor market is measured as the difference between the working hours in a paid activity before and after a family member becomes a caregiver translated in monetary terms. The goal is to estimate how much the working decisions of family members were affected by their caregiving duties. Nevertheless, as labor market participation does not only depend on personal decisions, but is also influenced by additional factors, some endogenous and others exogenous, we adjusted our estimates by the characteristics of the caregiver and the cared-for person. As for the characteristics of the family caregiver, we adjusted our estimates by gender, age group, education level, health status, partnership status, children in the household, and household income. As for the characteristics of the cared-for person, we adjusted our estimates by the level of the injury, i.e., paraplegia or tetraplegia, their level of dependency, and their working status.

## Results

### Descriptive statistics

We limited our study to family caregivers who were in working age, which gave us a total sample of 432 (N), see Table 1. Of them, 76% were female, with an average age of 50 years old. On average, participants started their role as caregivers when they were 39 years old. Our sample is composed of long-term caregivers, who have on average, spent almost 11 years caring for a relative. The average household size in the sample is 2 persons, and only 19% reported children living in the household. Family caregivers reported to be highly satisfied with their health, 7.5 on a 1–10 scale, where 10 stands for “very satisfied.”

**Table 1:** Descriptive statistics of family caregivers in working age

	Mean (%)	Std. Dev
Female	76%	
Age in years	49.92	10.26
Swiss nationality	88%	
In partnership	75%	
Satisfaction with health	7.46	2.15
Household size	1.97	1.19
Household with children	19%	
**Education:**		
No mandatory	3%	
Mandatory school	20%	
Secondary school	48%	
Higher education	29%	
**Household income**		
< 4500 CHF	17%	
4500—7500 CHF	40%	
> 7500 CHF	43%	
Financial satisfaction	6.78	2.5
**Working status**		
Has a paid work	75%	
Working pensum	61.6%	32.24
Unemployed	6%	
**Caregiving characteristics**		
Cared-for person is the partner	74%	
Years as caregiver	10.91	9.24
Has professional care support	30%	
**Characteristics cared-for person**		
Traumatic SCI	78%	
Completely dependent	74%	
Paraplegic	64%	
Cared-for person works	42%	
**N**	432	

Close to 77% of family caregivers had education above secondary level. Close to 40% reported a household income between CHF 4,500—7,500, which is around the median income at the country level [34]. Interestingly, 43% of participants reported a household income above CHF 7,500. On average, family caregivers reported a financial satisfaction close to 7, on a 1–10 scale, where 10 stands for “very satisfied.” As for labor market participation, in general, family caregivers were active in the labor market as 75% of them reported having a paid activity. Nonetheless, most of them worked part-time, 26 h per week—60% of a full-time job. Few of them (6%) reported being unemployed.

As for the person with SCI, or cared-for person, about 78% reported having SCI due to a traumatic event, e.g., car accidents, sports accidents, or falls, among others. A large share of the sample (74%) reported to be completed depended, 30% received professional home care and 42% of them reported having a paid work.

### Estimated forgone income

**Fig. 1:**
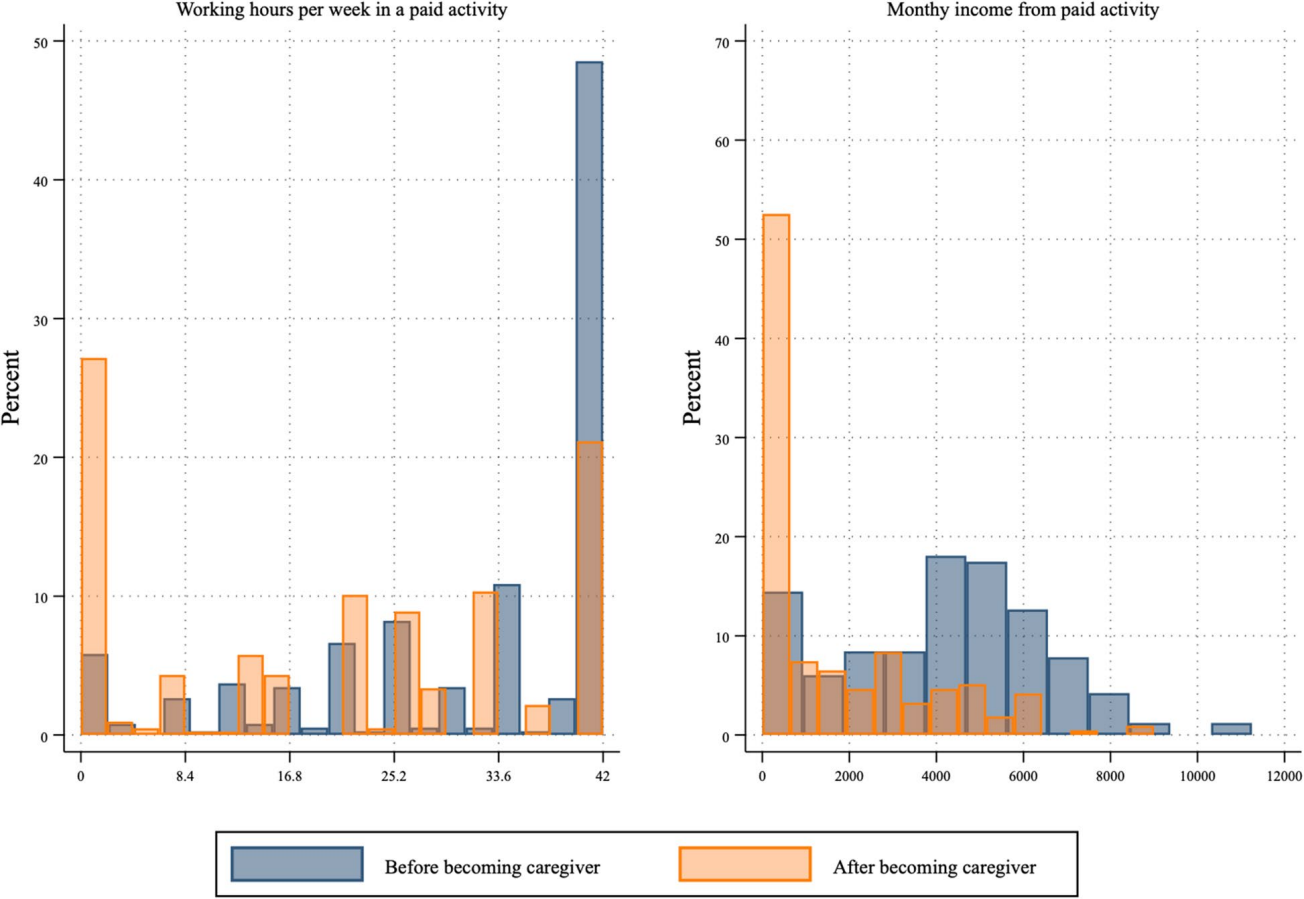
Opportunity costs in the labor market for family caregivers

Figure 1 summarizes the shifts in the labor market for family caregivers in hours and in monetary terms. The results show a marked decrease in working hours once family members become caregivers, which has a significant decrease in their salary. The changes are specially marked among the full-time workers, who before becoming caregivers (blue bars), were close to 50% of the sample, and after becoming caregivers (orange bars), decreased to around 20%. If disaggregated by gender, this shift is more marked among female caregivers, older caregivers, and caregivers who support persons facing higher levels of disability.

Table 2 reports the estimated effects in the labor market in working hours per week, and in monthly income adjusted by the characteristics of the caregiver and the cared-for person. In both cases, the results are reported in percentual changes, and in total values. The results describe how much family members have given up in the labor market to undertake their role as caregivers. On average, in the sample, family members reduced their working pensum by about 1 working day per week (8.4 h). This working time reduction had an average reduction in their income of CHF 970.8. The effect is more pronounced for women with a working time reduction of 8.68 h vs. 7.43 h for male caregivers, which translates in an income loss of about CHF 994.65 for women vs. CHF 897.25 for men.

**Table 2:** Estimated reduction in working hours and income for family caregivers

	**Working pensum: hours/week**				**Montly salary**			
	In percetange		In hours/week		In percentage		In monthly salary	
	**Mean**	**SD**	**Mean**	**SD**	**Mean**	**SD**	**Mean**	**SD**
**Total**	-23.0%	0.19	-8.37	5.74	-19.0%	0.17	- 970.77 CHF	778.72
**Caregiver characteristics**								
Female	-25.0%	0.19	-8.68	5.74	-21.0%	0.16	- 994.65 CHF	788.62
Male	-15.0%	0.18	-7.43	5.67	-13.0%	0.16	- 897.25 CHF	746.96
Age: 18–29 years old	-11.0%	0.17	-4.48	5.42	-8.0%	0.15	- 367.42 CHF	756.83
Age: 30–44 years old	-15.0%	0.17	-6.11	4.98	-12.0%	0.15	- 785.40 CHF	695.33
Age: 45–54 years old	-21.0%	0.18	-8.07	5.56	-19.0%	0.17	- 1057.01 CHF	784.71
Age: 55–64 years old	-29.0%	0.19	-10.17	5.64	-24.0%	0.16	- 1070.99 CHF	772.71
Education: lesss than mandatory	-24.0%	0.18	-10.13	5.2	-18.0%	0.16	- 1137.31 CHF	721.64
Education: mandatory	-25.0%	0.18	-9.77	5.03	-22.0%	0.15	- 1108.42 CHF	718.61
Education: secondary	-25.0%	0.19	-8.65	5.88	-20.0%	0.17	- 985.48 CHF	801.75
Education: higher	-18.0%	0.19	-6.82	5.7	-16.0%	0.16	- 839.04 CHF	771.89
**Cared-for person characteristics**								
SCI: Traumatic	-24.0%	0.19	-8.62	5.76	-20.0%	0.17	- 1017.03 CHF	789.66
SCI: Non-traumatic	-19.0%	0.19	-7.47	5.63	-15.0%	0.16	- 803.76 CHF	723.05
Paraplegia	-13.0%	0.14	-5.74	4.58	-10.0%	0.11	- 548.57 CHF	537.12
Tetraplegia	-41.0%	0.13	-13.15	4.4	-36.0%	0.11	- 1736.62 CHF	522.35
Completely dependent on wheelchair	-25.0%	0.18	-8.94	5.5	-21.0%	0.16	- 1042.02 CHF	758.34
Able to stand	-14.0%	0.25	-6.88	7.12	-12.0%	0.2	- 789.30 CHF	920.04
Partially able to walk	-15.0%	0.2	-6.49	6.04	-13.0%	0.17	- 730.06 CHF	791.2
Person SCI works	-15.0%	0.16	-6.08	4.89	-13.0%	0.14	- 651.11 CHF	675.63

Similarly, older caregivers decreased their working hours more compared to younger caregivers. While young caregivers reduced an average of 4.5 h per week of their working schedule, older caregivers reduced it by about 10.2 h per week. This translated into CHF 367.42 less income for the young group, compared to CHF 1070.9 less for the eldest group. Caregivers with higher education reduced by less their working hours (6.8 fewer hours per week, or CHF 839), compared to caregivers with fewer years of education (10.13 fewer hours per week, or CHF 1137.3).

As for the characteristics of the cared-for person; on average, caring for a person who suffered from a traumatic event, for a person with tetraplegia, and for a person completely dependent has the highest impact on the labor market for family caregivers. Interestingly, when the cared-for person reported working, the effect on the working time of family caregivers was significantly reduced (6.08 fewer hours per week, or CHF 651.1).

## Discussion

To undertake the role as caregivers, family members of persons with SCI reduce their working hours by about 23%, or 8.4 h per week. This represents a decrease in income of CHF 970.8 per month (CHF 11,650 per year), with significant variation by the caregivers’ characteristics. When disaggregating the results, female caregivers, older caregivers, and caregivers with lower education reduced by more their working hours, as a clear indication that their opportunity cost in the labor market is smaller, i.e., men, younger and higher educated family members have, on average, higher incomes, which makes it more costly for them to reduce their working hours. Also, caregivers of persons with tetraplegia experience the highest decrease in their working hours (40% reduction), with a reduction in their income of about CHF 1736. Even when the analysed data was collected between 2017-2018, we consider our estimates hold today because the macroeconomic factors associated with the Swiss labor market, such as inflation and unemployment rates, have been quite stable [35]. Nevertheless, if interest rates hikes and positive inflation become a common reality, the estimated income losses would be adjusted. 

Compared to related studies, our results show that in Switzerland, highly burdened caregivers, as is the case of our sample, are less likely to quit the labor market. Instead, family members are more likely to reduce their working time to undertake their role as caregivers. This is reflected in the small unemployment rate reported in the sample, but also in the number of family members that reported that caregiving did not affect their working conditions. This result has potentially two explanations, one is given by the characteristics of the Swiss labor market, where part-time jobs are common, especially among women. In fact, after the Netherlands, Switzerland is the country with the highest number of people employed in part-time jobs in Europe [36]. The second explanation is given by the range of possibilities Swiss employers offer to employees to combine working with caring time, like unpaid holidays, compensation times, caring days, among others. Interestingly, after the COVID-19 pandemic, this observed flexibility of the Swiss labor market is being expanded to groups and to areas where it was unusual. In fact, remote work is rapidly increasing, as well as part-time work, especially among men [35]. This change may have important implications for those households with caregiving responsibilities, as there would be more than one person in the household who can undertake with caring responsibilities. Nevertheless, even when this change is important, significant shifts on the share of caregiving tasks, and how much this will impact the participation in the labor market of family caregivers need more time to be evaluated. 

From a societal perspective, the effects in the labor market also represent a productivity loss for society, and should be contrasted to how much it would cost to replace the work of family caregivers with a market substitute. In this way it is possible to estimate what is optimal for society, and identify potential areas of action in the long-term care law. In fact, a parallel study, using the same sample, estimated that replacing the work undertaken by family caregivers with professional home care would cost, on average, CHF 5,644 per month (CHF 62,732 per year) [27]. This value is 5 times higher than the estimated productivity loss in the labor market, which implies that family caregivers represent important savings for the society [30]. In fact, in the absence of family caregivers, it is very unlikely the Swiss health and social system can cope with the increasing needs of care as the rise of people facing disability and requiring care is estimated to dramatically increase.

Nevertheless, our estimates should be taken carefully as additional costs and benefits of caregiving are not included in the valuation method [20]. The estimated opportunity cost can be seen as an underestimation of the total value of the work undertaken by family caregivers, as we only considered paid work, and disregarded leisure time, and unpaid work, which are the areas that family members reduce to take over their caregiving duties [19, 22, 37]. In fact, our results show that family members do not reduce their working time 1:1 compared to caregiving time. The reduction in working hours is three times smaller than the reported average caregiving time (27 h per week). This implies family members add caring responsibilities to their existing work, with potentially detrimental effects on their health over time. In fact, overly burdened caregivers are more likely to give up their caregiving duties, and send their family members to nursing homes, a situation that is not always optimal for the cared-for person.

Thus, health and social systems need to find ways to support family caregivers as substitution of their work is not desirable neither from the financial perspective nor for the wellbeing of the cared-for persons [38]. In this matter, there are at least two important points to consider. The first one is the immediate urgency that economic evaluations include the role of family caregivers to ensure that their needs are taken into consideration. Some systems support the role of family caregivers through cash reimbursements for care activities, counseling, and complementary professional home support [39], also, some systems offer the provision of retirement funds and career benefits [40].

Finally, a more long-term strategy is for the health and social systems to shift their focus to the provision of rehabilitation for persons facing disability. Rehabilitation aims at improving functioning, which also implies that people become more independent. Therefore, rehabilitation, while directly targeting the person facing disability, also improves the living situation of family caregivers, as the needs of care are reduced. More investment and research in rehabilitation could improve the quality of life not only for the individual with SCI but also for their caregivers, leading to more productivity for the economy in general.

## Limitations

Some limitations are worth mentioning in light of the results. First, because of the lack of data, some assumptions were made to calculate the salaries for family members who worked before becoming caregivers. All the assumptions followed a conservative approach; thus, our results are likely to underestimate the estimated opportunity cost. Second, the questionnaire targeted only the primary family caregivers and excluded all other sources of support from other family members. This again implies our results may be an underestimation of the opportunity cost in the labor market, as other family members may have also been affected in their working decisions to support the person in need of care.

In addition, the questionnaire had a total of 138 items, some of which required detailed information. The length and complexity of the questionnaire may have discouraged caregivers experiencing high levels of burden, a group that may also face the most drastic effects in the labor market. Moreover, the questionnaire was available in the three Swiss official languages, German, Italian and French, which may have excluded an important number of non-Swiss caregivers. In fact, Switzerland has an important number of immigrants whose mother tongue is none of the official languages.

In reference to the opportunity cost method. On the one hand, it only considers the forgone income of family caregivers and disregards the benefits that being a caregiver can convey, for example, the better relationship with the care recipient and the positive feelings that helping a loved one entails [9]. On the other hand, in this study, the opportunity cost does not consider the leisure and personal care time that is displaced because of the care activities of the primary family caregivers and of other family members providing care [41]. This reduction in self-care time could lead to negative health effects, for example, less sleep time [20], bodily pain, fatigue and less vitality, which translated into increasing health care costs for family caregivers [42]. Additionally, this method underestimates the value of the work performed by women and elderly because of lower wages in the labor market [21].

Despite all of the mentioned limitations, this study provides a valid approximation of the costs that SCI primary family caregivers have in Switzerland. Even though it is an approximation, it is useful to have a monetary estimation of the costs that should be considered instead of taking informal care as a free or voluntary resource [38]. In our study, however, we obtained information on the work situation before and after being a caregiver for the same person, and we compared the same caregiver at two different times. Due to the difficulty in obtaining this information, most related research depends on income estimates based on educational levels, average salaries, and the patient’s severity; in this study, however, we were able to use the reported caregiver wages, which is a much accurate approximation.

## Conclusion

Family caregivers of persons with SCI reduced, on average, 23% of their working time to undertake their role as caregivers. This is almost 4 times fewer hours than the time family members spent on caregiving duties. This implies family members put caregiving tasks on top of their existing responsibilities, with potential detrimental effects on the health and well-being of themselves and the cared-for person. Thus, the estimated numbers should be seen as an underestimation of the total value of the work performed by family caregivers.

From a societal perspective, the role family caregivers play in long-term care translates into important savings for the health and social systems as professional home care is expensive. Thus, replacing their work is neither optimal nor sustainable. Nevertheless, it is essential to find ways to support and compensate them. The economic evaluations of their work provide a range to define compensation measures to keep family members involved in the caregiving process when desirable. The estimated income loss can be seen as a lower bound reference for compensation, and the cost of a market substitute, the upper bound reference for compensation.

## Data Availability

The data that support the findings of this study are available from Swiss Paraplegic Research (SPF) but restrictions apply to the availability of these data, which were used under license for the current study, and so are not publicly available. Data are however available from the corresponding author upon reasonable request and with permission of SPF.

## Abbreviations

SCI: Spinal cord injury
NOGA: General Classification for Economic Activities
FSO: Federal Statistical Office

## References

[1] van den Berg B, Brouwer WBF, Koopmanschap MA (2004). Economic valuation of informal care. An overview of methods and applications. Eur J Health Econ.

[2] Arksey H. Combining informal care and work: supporting carers in the workplace. Health Soc Care Community. 2002;10(3):151–61. 10.1046/j.1365-2524.2002.00353.x. 10.1046/j.1365-2524.2002.00353.x 12121251

[3] Bauer J, Sousa-Poza A. Impacts of Informal Caregiving on Caregiver Employment, Health, and Family. Popul Ageing. 2015;8:113–145. 10.1007/s12062-015-9116-0.

[4] Goodrich K, Kaambwa B, Al-Janabi H (2012). The inclusion of informal care in applied economic evaluation: a review. Value Health.

[5] Lilly MB, Laporte A, Coyte PC. Labor market work and home care's unpaid caregivers: a systematic review of labor force participation rates, predictors of labor market withdrawal, and hours of work. Milbank Q. 2007;85(4):641-90. 10.1111/j.1468-0009.2007.00504.x. 10.1111/j.1468-0009.2007.00504.x PMC2690351 18070333

[6] Colombo F, et al. Help Wanted?: Providing and Paying for Long-Term Care, OECD Health Policy Studies, OECD Publishing, Paris; 2011. 10.1787/9789264097759-en.

[7] Hoffmann F, Rodrigues R. Informal Carers: Who Takes Care of Them? Policy Brief 4/2010. Vienna: European Centre; 2010.

[8] Gemperli A, Rubinelli S, Zanini C, Huang J, Brach M, Pacheco Barzallo D. Living situation of family caregivers of persons with spinal cord injury. J Rehabil Med. 2020;52(11):jrm00124. 10.2340/16501977-2762. 10.2340/16501977-2762 33119123

[9] Charlifue SB, Botticello A, Kolakowsky-Hayner SA, Richards JS, Tulsky DS (2016). Family caregivers of individuals with spinal cord injury: exploring the stresses and benefits. Spinal Cord.

[10] Singh A, Tetreault L, Kalsi-Ryan S, Nouri A, Fehlings MG (2014). Global prevalence and incidence of traumatic spinal cord injury. Clin Epidemiol.

[11] Kang Y, Ding H, Zhou HX, Wei ZJ, Liu L, Pan DY, Feng SQ. Epidemiology of worldwide spinal cord injury: a literature review. J Neurorestoratology. 2018;6:1–9. 10.2147/JN.S143236.

[12] Yoon J, Chee CP, Su P, Almenoff P, Zulman DM, Wagner TH. Persistence of High Health Care Costs among VA Patients. Health Serv Res. 2018;53(5):3898–916. 10.1111/1475-6773.12989. 10.1111/1475-6773.12989 PMC6153161 29862504

[13] Huang J, Pacheco Barzallo D, Rubinelli S, et al. What influences the use of professional home care for individuals with spinal cord injury? A cross-sectional study on family caregivers. Spinal Cord. 2019;57:924–32. 10.1038/s41393-019-0296-y. 10.1038/s41393-019-0296-y PMC6892416 31127196

[14] Tikkanen R, Osborn R, Mossialos E, Djordjevic A, Wharton GA. International health care system profiles. The Commonwealth Fund [Internet]. 2020.

[15] Donelan K, Hill CA, Hoffman C, Scoles K, Feldman PH, Levine C, Gould D. Challenged to care: informal caregivers in a changing health system. Health Aff (Millwood). 2002;21(4):222–31. 10.1377/hlthaff.21.4.222. 10.1377/hlthaff.21.4.222 12117133

[16] Gibson MJ, Houser A. Valuing the invaluable: a new look at the economic value of family caregiving. Issue Brief (Public Policy Inst (Am Assoc Retired Pers)). 2007;(IB82):1–12. 17612038

[17] Ansah JP, Matchar DB, Malhotra R, Love SR, Liu C, Do Y. Projecting the effects of long-term care policy on the labor market participation of primary informal family caregivers of elderly with disability: insights from a dynamic simulation model. BMC Geriatr. 2016;16:69. 10.1186/s12877-016-0243-0. 10.1186/s12877-016-0243-0 PMC4806512 27007720

[18] The Lancet Healthy Longevity. Care for ageing populations globally. Lancet Healthy Longev. 2021;2(4):e180. 10.1016/S2666-7568(21)00064-7. 10.1016/S2666-7568(21)00064-7 PMC8529576 34697611

[19] Oliva-Moreno J, Trapero-Bertran M, Peña-Longobardo LM, Del Pozo-Rubio R. The Valuation of Informal Care in Cost-of-Illness Studies: A Systematic Review. Pharmacoeconomics. 2017;35(3):331–45. 10.1007/s40273-016-0468-y 27848219

[20] Grosse SD, Pike J, Soelaeman R, Tilford JM (2019). Quantifying Family Spillover Effects in Economic Evaluations: Measurement and Valuation of Informal Care Time. Pharmacoeconomics.

[21] Paraponaris A, Davin B (2015). Economics of the Iceberg: Informal Care Provided to French Elderly with Dementia. Value Health.

[22] van den Berg B, Brouwer W, van Exel J, Koopmanschap M, van den Bos GAM, Rutten F (2006). Economic valuation of informal care: lessons from the application of the opportunity costs and proxy good methods. Soc Sci Med.

[23] van Exel J, Bobinac A, Koopmanschap M, Brouwer W. The invisible hands made visible: recognizing the value of informal care in healthcare decision-making. Expert Rev Pharmacoecon Outcomes Res. 2008;8(6):557–61. 10.1586/14737167.8.6.557. 10.1586/14737167.8.6.557 20528366

[24] Committee on Family Caregiving for Older Adults; Board on Health Care Services; Health and Medicine Division; National Academies of Sciences, Engineering, and Medicine. Families Caring for an Aging America. Schulz R, Eden J, editors. Washington (DC): National Academies Press (US); 2016. 27905704

[25] Pletscher M, Mattli R, Reich O, Von Wyl A, Wieser S. The Societal Costs of Schizophrenia in Switzerland. Value Health J Int Soc Pharmacoeconomics Outcomes Res. 2014;17(7):A457. 10.1016/j.jval.2014.08.1253 27201274

[26] Kraft E, Marti M, Werner S, Sommer H (2010). Cost of dementia in Switzerland. Swiss Med Wkly.

[27] Pacheco Barzallo D, Hernandez R, Brach M, Gemperli A. The economic value of long-term family caregiving. The situation of caregivers of persons with spinal cord injury in Switzerland. Health & Social Care in the Community [Internet]. [cited 2022 May 26];n/a(n/a). Available from: https://onlinelibrary.wiley.com/doi/abs/10.1111/hsc.13668. 10.1111/hsc.13668 PMC9543297 34854509

[28] Mudrazija S (2019). Work-Related Opportunity Costs of Providing Unpaid Family Care In 2013 And 2050. Health Aff.

[29] Wilcox, V., Sahni, H. The Effects on Labor Supply of Living with Older Family Members Needing Assistance with Activities of Daily Living. J Fam Econ Iss. 2022. 10.1007/s10834-022-09880-x. 10.1007/s10834-022-09880-x PMC9748888 36533120

[30] Chari AV, Engberg J, Ray KN, Mehrotra A (2015). The Opportunity Costs of Informal Elder-Care in the United States: New Estimates from the American Time Use Survey. Health Serv Res.

[31] Ruiz-Adame Reina M, Correa M, Burton K (2019). The opportunity costs of caring for people with dementia in Southern Spain. Gac Sanit.

[32] Post MW, Brinkhof MW, von Elm E, Boldt C, Brach M, Fekete C, Eriks-Hoogland I, Curt A, Stucki G; SwiSCI study group. Design of the Swiss Spinal Cord Injury Cohort Study. Am J Phys Med Rehabil. 2011;90(11 Suppl 2):S5–16. 10.1097/PHM.0b013e318230fd41. 10.1097/PHM.0b013e318230fd41 21975676

[33] Statistik B für. Salarium – Statistischer Lohnrechner [Internet]. [cited 2022 May 26]. Available from: https://www.bfs.admin.ch/bfs/de/home/statistiken/arbeit-erwerb/loehne-erwerbseinkommen-arbeitskosten/lohnniveau-schweiz/salarium.html.

[34] Office fédéral de la statistique, Enquête sur le budget des ménages (EBM) Renseignements: Enquête sur le budget des ménages, ebm@bfs.admin.ch, tél. 058 467 25 83© OFS – Encyclopédie statistique de la Suisse.

[35] Federal Statistical Office. Labour market indicators for 2021 [Internet]. Neuchatel, Switzerland; 2021 Aug. (Work and Income). Report No.: FSO News. Available from: https://www.swissstats.bfs.admin.ch/collection/ch.admin.bfs.swissstat.en.issue21032062106/article/issue21032062106-01.

[36] OECD. Part-time employment rate (indicator). 2023. 10.1787/f2ad596c-en.

[37] Riewpaiboon A, Riewpaiboon W, Ponsoongnern K, Van den Berg B (2009). Economic valuation of informal care in Asia: a case study of care for disabled stroke survivors in Thailand. Soc Sci Med.

[38] Muurinen JM (1986). The economics of informal care. Labor market effects in the National Hospice Study. Med Care.

[39] Estrada Fernández ME, Gil Lacruz AI, Gil Lacruz M, Viñas López A. Informal care. European situation and approximation of a reality. Health Policy. 2019;123(12):1163–72. 10.1016/j.healthpol.2019.09.007. 10.1016/j.healthpol.2019.09.007 31606144

[40] van BroeseGroenou MI, De Boer A (2016). Providing informal care in a changing society. Eur J Ageing..

[41] Pacheco Barzallo D. Spillover Effects of Long-Term Disabilities on Close Family Members. Appl Health Econ Health Policy. 2018;16(3):347–55. 10.1007/s40258-018-0391-9. 10.1007/s40258-018-0391-9 29651776

[42] Blanes L, Carmagnani MIS, Ferreira LM (2007). Health-related quality of life of primary caregivers of persons with paraplegia. Spinal Cord.

